# Supplementary material for: Comparison of whole-body muscle imaging findings between GNE myopathy and other young adult-onset hereditary myopathies
Source: PLoS One. 2026 Jan 23;21(1):e0341031. doi: 10.1371/journal.pone.0341031 (PMC12829776; doi:10.1371/journal.pone.0341031)
Supplement: S2 File — (DOCX) [file pone.0341031.s002.docx]

**Supplementary materials**

**Study protocol**

This retrospective cohort study was approved by the ethics committee of the Faculty of Medicine, Prince of Songkla University (REC. 66-539-14-3). Data were retrieved from a neuromuscular clinical registry at a university-based hospital, a major referral center covering 14 provinces in southern Thailand, between 2019 and 2024. The inclusion criteria were: 1) diagnosis of GNE myopathy (confirmed by biallelic pathogenic variants of GNE) or young adult-onset hereditary myopathies with limb girdle weakness pattern (confirmed by either genetic tests, muscle biopsies, or MRI), and 2) available primary whole-body MRI imaging data.

Whole-body MRI was performed using a 1.5-T scanner (Philips Medical Systems, Best, Netherlands). The anatomic coverage extended from the skull base to both ankles and from the anterior to posterior surface of the body, in a head-first direction. Depending on patient height, scans were divided into 4–5 contiguous sections (MRI stations). The following pulse sequences were used: coronal T1W Dixon images (including in-phase, opposed-phase, water-only and fat-only), repetition time 400 ms, echo time 13 ms, field of view 265 mm, matrix 256×112; axial T1WI, repetition time 475 ms, echo time 10 ms, field of view 350 mm, matrix 512×272; and short tau inversion recovery (STIR) imaging, repetition time 1500 ms, echo time 15 ms, field of view 350 mm, matrix 512×272. Contrast medium was not used in any of the cases. Additionally, axial quantitative proton-density fat-fraction (PDFF) measurements were performed using the mDIXON Quant technique.

Whole-body MRI parameters were evaluated into two domains. First, in the anatomic domain, whole-body muscles were classified into the following six regions: 1) the cranium and neck (temporalis, masseter, medial pterygoid, lateral pterygoid, tongue, sternocleidomastoid, cervical extensor, and longus collis), 2) shoulder girdle (latissimus dorsi, trapezius, deltoid, supraspinatus, infraspinatus, subscapularis, pectoralis minor and major, and serratus anterior), 3) body and upper limbs (biceps, triceps, anterior compartment of the forearm [flexor group], mobile-wad muscles of the forearm [brachioradialis, extensor carpi radialis longus and brevis], posterior compartment of the forearm [extensor group], intercostal muscles, thoracic extensor, lumbar extensor, psoas muscle and abdominal belt muscles), 4) pelvis (gluteus maximus, gluteus medius, gluteus minimus, adductor magnus, adductor longus and pectineus), 5) thighs (rectus femoris, vastus lateralis, vastus intermedius, vastus medialis, sartorius, gracilis, semitendinosus, semimembranous, biceps femoris long head and biceps femoris short head), and 6) lower legs (gastrocnemius medialis, gastrocnemius lateralis, soleus, tibialis anterior, tibialis posterior, extensor hallucis longus and extensor digitorum longus, flexor digitorum longus and peronei).

The second domain involved grading abnormalities in each MRI sequence, including T1-weighted, STIR, and Dixon images (Figure 1). T1WI aimed to detect the fatty tissue replacement which was classified into five grades [1]: grade 0, normal appearance; grade 1, mild condition with trace amounts of fat signal (<25%); grade 2, moderate condition with initial confluence observed in <50% of muscle; grade 3, severe condition with confluence observed in >50% of muscle; grade 4, end-stage disease with fat signals covering the entire muscle. STIR was used to determine edema or active inflammation, graded as present or absent (Figure 1). In the mDIXON Quant technique used to determine the percentage of fat replacement, PDFF was measured by selecting a circular region of interest (ROI) approximately 50 mm2 in area in the central region of each muscle. Fat measurements in the periphery of the images may be limited in patients with large body habitus due to artifacts. To standardize the sampling of the muscle area in PDFF measurements, the specific axial view in mDIXON-QUANT was set. The specific axial view cut and landmark in the thigh and lower leg regions were set according to a previously reported protocol [2]. For regions beyond the lower limbs, no well-defined landmarks have been reported; therefore, we specified the following landmarks: 1) cranium (axial view cut at the mandibular angle for evaluating lateral pterygoid and masseter) and neck (axial view cut at C4 the transverse process for evaluating cervical extensors), 2) shoulder girdle (axial view cut at the level of the pectoralis major muscle insertion at the humerus for evaluating the deltoid, subscapularis, and infraspinatus muscles, and axial view cut at the level of the inferior scapular tip for evaluating the latissimus dorsi and serratus anterior), 3) body (axial view cut at the level of the inferior scapular tip for evaluating thoracic extensors, and axial view cut at the L3 transverse process for evaluating psoas and lumbar extensors [3]) and upper limbs (axial view cut at the insertion of the deltoid muscles for evaluating the biceps and triceps, and axial view cut at the insertion of the extensor carpi radialis brevis for evaluating anterior and posterior compartments of the forearm), and 4) pelvis (axial view cut at the level of the pelvis, near the sciatic foramen, for evaluating the gluteus maximus, gluteus medius, and gluteus minimus). MRI parameter was collected independently by two musculoskeletal radiologists (P.T. and P.B.), and any discordant parameters were resolved through discussion until a consensus was reached.

The clinical data included demographic data, genetic test results, and functional scores evaluated within 1 month before or after MRI. The Brooke and Vignos scale [4] were used in both groups to assess upper and lower extremity function. Additionally, the GNE myopathy functional activity scale (GNEM-FAS) was used as a disease-specific measurement. In this scale, each item is rated from 0 to 4, with higher scores representing better function; the total score ranged from 0 to 100, comprising 40 points for mobility, 32 points for upper extremities function, and 28 points for self-care [5].

Descriptive demographic and clinical data are presented as numbers and percentages for discrete data and means ± standard deviations or medians (interquartile ranges) for continuous data. Detailed whole-body MRI findings from the GNE group are presented as a heat map indicating severity scores in each sequence. Whole-body MRI findings were compared between groups using the chi-squared test, independent t-test, or Mann–Whitney U test. Inter-rater reliability was examined using Cohen’s kappa coefficient.

Reference

1. Fischer D, Kley RA, Strach K, Meyer C, Sommer T, Eger K, et al. Distinct muscle imaging patterns in myofibrillar myopathies. Neurology. 2008;71: 758–765. doi: [10.1212/01.wnl.0000324927.28817.9b](https://doi.org/10.1212/01.wnl.0000324927.28817.9b).

2. Veeger TTJ, Van De Velde NM, Keene KR, Niks EH, Hooijmans MT, Webb AG, et al. Baseline fat fraction is a strong predictor of disease progression in Becker muscular dystrophy. NMR Biomed. 2022;35: e4691. doi: [10.1002/nbm.4691](https://doi.org/10.1002/nbm.4691).

3. Tagliafico AS, Bignotti B, Torri L, Rossi F. Sarcopenia: How to measure, when and why. Radiol Med. 2022;127: 228–237. doi: [10.1007/s11547-022-01450-3](https://doi.org/10.1007/s11547-022-01450-3).

4. Lue YJ, Lin RF, Chen SS, Lu YM. Measurement of the functional status of patients with different types of muscular dystrophy. Kaohsiung J Med Sci. 2009;25: 325–333. doi: [10.1016/S1607-551X(09)70523-6](https://doi.org/10.1016/S1607-551X(09)70523-6).

5. Mayhew J, Bonner N, Arbuckle R, Turnbull A, Bowden A, Skrinar A. Development and preliminary evidence of the psychometric properties of the GNE myopathy functional activity scale. J Comp Eff Res. 2018;7: 381–395. doi: [10.2217/cer-2017-0062](https://doi.org/10.2217/cer-2017-0062).
