# Supplementary material for: Comparison of whole-body muscle imaging findings between GNE myopathy and other young adult-onset hereditary myopathies
Source: PLoS One. 2026 Jan 23;21(1):e0341031. doi: 10.1371/journal.pone.0341031 (PMC12829776; doi:10.1371/journal.pone.0341031)
Supplement: Attachment — Submitted filename: Response to reviewer.docx [file pone.0341031.s003.docx]

Response to reviewer

Dear Reviewers and staff editors,

Refer to the previous submission (PONE-D-25-06784)

Manuscript title: Comparison of whole-body muscle imaging findings between GNE myopathy and other young adult-onset hereditary myopathies

As the previous submitted manuscript (PONE-D-25-06784) raised the ethical issue concern, I believed that there was a misunderstanding in the method section due to the unclear statement. With permission after discussing this issue with the editorial assistant, I would like to address the response to reviewer’s comment as the following and resubmit this manuscript for the consideration.

| Reviewer’s comment | Response to reviewer’s comment | Change in text |
| --- | --- | --- |
| We recognize that you obtained ethical approval from your local ethics committee after you began the study: your ethics approval document is dated January 24, 2024, while personal data were collected and accessed beginning in 1st January 2019. For human subjects research, PLOS ONE requires that authors obtain ethics committee approval before beginning the study, and we do not accept retrospective ethical approval. | Regarding the ethical issue, this study has been approved by the ethical committee on January 24, 2024 which allowed us to retrospectively collect the data from the registry data dated back to January 1st, 2019. The date that we began data collection started after we have ethics committee approval.    I believed that the statement in the method section might not be clear enough which led to the misunderstanding.  We have clarified this statement in the method section as the following “This retrospective cohort study was approved by the ethics committee of the Faculty of Medicine, Prince of Songkla University (REC. 66-539-14-3), with the initial data access on 31st January 2024. The data from a neuromuscular clinical registry at a university-based hospital, a major referral center covering 14 provinces in southern Thailand, between 1st January 2019 and 31st December 2023 were retrospectively retrieved.” | File manuscript  Material and methods section  Page 3  Line 64-68 |

Thank you for your compassionate opportunity to allow us to explain and correct this unintentional misunderstanding.

Sincerely Yours,

Pat Korathanakhun, M.D.
