# Supplementary material for: Comparison of whole-body muscle imaging findings between GNE myopathy and other young adult-onset hereditary myopathies
Source: PLoS One. 2026 Jan 23;21(1):e0341031. doi: 10.1371/journal.pone.0341031 (PMC12829776; doi:10.1371/journal.pone.0341031)
Supplement: Attachment — Submitted filename: Response to reviewers comments.docx [file pone.0341031.s004.docx]

**Response to reviewers’ comments**

Dear Editor and reviewers,

Thank you for your valuable comments that sharpening our idea. I have thoroughly read the reviewers’ comments and edited the manuscript. The major comment effects the major change in the control group that showed heterogenous clinical and genetic result. According to the clinical practice, clinicians usually start with recognizing the distribution of muscle weakness. Therefore, we reset the control group by recruiting “young adult-onset hereditary myopathy with limb-girdle weakness pattern”.

We have explained the reason why the control group was selected in the introduction section as the following “Although GNE myopathy had a recognizable clinical course showing early foot drop followed by proximal arms and legs weakness, some patients, especially in case of longstanding inconclusive diagnosis, may present with late stage which all muscle were involved and the patient may not recognize the sequential symptoms accurately. In this setting, the differentiation GNE from other limb girdle pattern myopathy was very difficult and additional investigations are needed.”

Considering the article length, we optimized this issue by concision the method and refer all details to the supplementary data. We also merge the comparison data of T1WI grading and PDFF into a single figure (figure 2). In addition, the same abbreviations used in the figure 3 legend were shortening by referring to the abbreviation in the figure 2.

Due to the change of the control group, the results, figures and discussion were all edited. Please find the response to reviewers’ comments in the table below.

Sincerely Yours,

Pat Korathanakhun, M.D.

| **Reviewer #1:** The article is well written and the description of this rare entity is a valuable contribution to the scientific community.  However, the article is a little too long, and in my opinion, some parts should be removed to make it easier to read and avoid drowning out the main message:  - I don't believe that quantitative sequences are necessary  **Reviewer #2:**  Many data are similar between T1 and DIXON image analysis, so it is preferrable to eliminate repetitions and focus on muscle groups where DIXON images are more accurate for their best resolution. | This study aimed to explore the distinctive feature of GNE myopathy by using whole body muscles MRI. Initially, we would like to explore the clinical application of the mDIXON Quant image as a novel tool. However, the result turns identical to the simple grading by T1WI.  Therefore, we reorganized the data presenting by merge the comparison of fatty tissue replacement grade in T1WI and PDFF in mDIXON Quant in the figure 2 together with the descriptive fatty tissue replacement grade in T1WI among the GNE myopathy group.  Although the PDFF in mDIXON Quant imaging provide the better detail, the practical use is limited by several reasons. First, the limited resolution of mDIXON Quant image made it difficult to identify a clear muscle border in certain small muscle and the accurate measurement of PDFF parameter was impossible. Second, this method was labor and time consuming which was not suitable for clinical practice. Therefore, we choose to present the descriptive data of fatty tissue replacement grade in T1WI which was practical and offer acceptable accuracy.  Considering the word count, we also remove the abbreviation in the figure3 description and refer to the same abbreviation use in figure 2. | Figure 2 and result section “Comparison of T1WI and mDIXON Quant imaging between GNE and young adult-onset hereditary myopathies group”  Description of figure 3 |
| --- | --- | --- |
| Reviewer #1: - in view of the small number of myopathy cases I've noted typed for comparison, I don't think their description is necessary either a single fsh is not relevant for comparison with the GNE myopathy panel | We agree that the FSHD were not an appropriate subject in the control group.  we reset the control group by recruiting “young adult-onset hereditary myopathy with limb-girdle weakness pattern”.  We have explained the reason why the control group was selected in the introduction section as the following “Although GNE myopathy had a recognizable clinical course showing early foot drop followed by proximal arms and legs weakness, some patients, especially in case of longstanding inconclusive diagnosis, may present with late stage which all muscle were involved and the patient may not recognize the sequential symptoms accurately. In this setting, the differentiation GNE from other limb girdle pattern myopathy was very difficult and additional investigations are needed.” | Introduction  result and discussion |
| Reviewer #1: - a single descriptive analysis of your population already seems very relevant to me | Thank you |  |
| Reviewer #1: - to improve overall reading, a diagram with a color scale representing the frequency of muscles affected by anatomical region could be suggested | Thank you for your suggestion. We have tried to redesign the data visualization by many methods. We think that the updated version of the figure 2 would provide the good detail of GNE myopathy description and the comparison of parameters. | Figure 2 |
| Reviewer #2: The novelty of this study lies in the whole-body protocol, evaluating cranial and upper limb muscles, and in the partial homogeneity of the population (the common c.2179G>A variant). Nevertheless, I do not understand how the comparison group adds anything to the study and find numerous inaccuracies and redundant concepts.  The author claims that the inclusion criteria for the "control" group are young adult-onset hereditary myopathies (confirmed through genetic tests, muscle biopsies, or MRI). It is unclear whether some of the other myopathies used as controls have a genetic diagnosis. (e.g. generic limb-girdle muscular dystrophy?). If they do not, it must be indicated how the diagnosis of GNE has been excluded. Calpainopathy is an LGMD, why is it listed separately? It would also be useful to specify why those myopathies were chosen: typically, GNE has a foot drop onset (This is also acknowledged in the author’s discussion (p. 18 line 398)) and many other myopathies (e.g. distal early onset myopathies such as MYH7, TTN) are not considered. | We accepted that the control group has heterogeneous genotype and phenotype. We do agree that it would be perfect if the control group could confine only the distal myopathy which correlate to distal onset myopathy of GNE. However, there are 2 main reasons explaining why we could not do that.  First, the GNE myopathy patients in our registry were in the moderate to advanced disease stage which involving both proximal and distal muscles. With this clinical picture, it may also mimic other advanced stage hereditary myopathies.  Second, we do not have MYH7 and TTN patients in our registry.  For these reasons, we have tried to optimize choosing the proper control group based on the muscle involvement.  Although GNE myopathy had a recognizable clinical course showing early foot drop followed by proximal arms and legs weakness, some patients, especially in case of longstanding inconclusive diagnosis, may present with late stage which all muscle were involved and the patient may not recognize the sequential symptoms accurately. In this setting, the differentiation GNE from other limb girdle pattern myopathy was very difficult.  Therefore, we reset the control group by recruiting “young adult-onset hereditary myopathy with limb-girdle weakness pattern”.  For the detail of control group, we edited as the following:  “’ Ten patients with young adult-onset hereditary myopathies with limb-girdle weakness pattern consisted of 4 genetically confirmed dystrophinopathy patients and 6 limb-girdle muscular dystrophy patients (2 genetically confirmed calpainopathy patients, 1 genetically confirmed dysferlinopathy and 3 patients with clinicopathological correlation with limb-girdle muscular dystrophy (LGMD) and having negative result for comprehensive neuromuscular gene panel test (211 genes testing provided by INVITAE company))”  And also in the  Strengths, limitations, clinical implication and suggestions section as the following:  “ There are some concerns for using this research data in the clinical practice. In general approach, the clinician always start the clinical approach of hereditary myopathy by the distribution of the affected part in which GNE myopathy started with distal myopathy. However, in case of late presentation with advanced disease stage, the affected parts blended both proximal and distal muscle. The finding in this research would guide some clinical clues to recognize GNE myopathy in case of late presentation as the population in this study were in the moderate to advanced disease stage. The comparion study of early GNE myopathy with other distal myopathy will be the area of future interest. In addition, muscle imaging research in GNE myopathy should expand the imaging protocol to include more body regions.” | Introduction  Method  Result section  Strength, limitations, clinical implication and suggestions section |
| In fact, in the discussion paragraph "Variations in findings across MRI sequences" there is absolutely no comparison between the two techniques and it is not clear why I should prefer either one or the other. | We change the subtopic from "Variations in findings across MRI sequences" to “The practical use of T1WI, mDIXON Quant and STIR “    We added this issue in the discussion as the following:  “The fatty tissue replacement grading in T1WI is a simple visual scale method and offers a reliable result confirm by good interrater reliability in our study. The detail of the fatty tissue replacement grade was sufficient for clinical practice. Although the mDIXON Quant image provide the greater quantitative detail, several ltechnical imitations were considered. First, this method is labor and time consuming. Second, the PDFF was measure in the focal area specified in the standardized axial cut of each muscle. With this technic, the sampling error might occur in the muscle that had scattered patchy fatty tissue replacement while the visual scale grading in T1WI evaluate the whole muscle mass. Third, due to the limited mDIXON Quant image resolution, the accuracy of muscle border identification, especially in the small muscle, was inconsistent which increase the possibility of data collection error. To our experience, we would suggest that the visual scale grading in T1WI was reliable method and offer sufficient data for clinical practice, while the quantitative PDFF in mDIXON Quant image was suitable for the research purpose. “ | Discussion section  Subtopic “The practical use of T1WI, mDIXON Quant and STIR“  Page 13 line 278 – page 14 |
| In lower limb analysis, it is well known that Biceps femoris short head is the first muscle involved in GNE and is therefore configured as a pathognomic for diagnosis along with quadriceps sparing. By not differentiating the two ends of the femoral biceps in the analysis, the author loses an important muscle, if not the pivotal muscle, able to differentiate this pathology from the others included. It is indeed surprising that a difference was NOT identified between the two groups in the thigh section. | Thank you for your sharp comment. We collect additional data in short head and long head biceps femoris as suggested. The data were shown in Figure 2 and 3. Finally we found that the short head biceps femoris in GNE myopathy was more affected than those in other young adult-onset hereditary myopathy. | Figure2 and 3  Result section “Comparison of T1WI and mDIXON Quant imaging between GNE and young adult-onset hereditary myopathies group” |
| ":Herein, in patients with GNE with GNEM-FAS scores indicative of more severe disease, pathological changes were seen in advanced stages in the vastus lateralis on STIR imaging". This sentence is not supported by the data reported in the results | This part was clarified as the following:  “Herein, vastus lateralis muscle edema were observed in the advanced stage GNE myopathy patient. This is consistent with the sequential involvement of quadriceps in which the vastus lateralis is affected later than the vastus medialis, vastus intermedius, and rectus femoris. In addition, in the shoulder girdle, STIR revealed pathological changes in the supraspinatus and infraspinatus muscles in advanced GNE myopathy.” | Discussion  Page 14, Line 297-302 |
| p 17 line 389-391 the author should comment on the results of his study and comparisons with the "small" control group e.g., the obturator internus which is mentioned here, does not appear among the muscles analyzed by the author nor among those significant. The comparison with FSHD (there is only one FSHD in the comparison group) or myofibrillar myopathies (they are not present in the comparison group) is mentioned several times in the discussion. The differential diagnosis with LGMD that would have led the author to include them is never addressed | As the control group was change into the “young adult-onset hereditary myopathy with limb girdle weakness pattern”, the control group were comprised of Dystrophinopathies patients and LGMD patients. Therefore, the discussion part were changed by discussion based on the dystrophinopathies and LGMD data. | Discussion  Page 15-16 |
| p 17 line 392, ST involvement is typical of ONE myofibrillar myopathy = desminopathy, and also an early feature of HMERF (10.1016/j.nmd.2017.12.002) |  |  |
| In all this discussion It’s not clear what the author means with "the correlation between disease severity and certain muscles" |  |  |
| please use either QUANT or quant in the text | Sorry for typing error. We have corrected this issue. |  |
| pag 6 line 122 PDFF should de spelled also in the text not only in the figure | Sorry for typing error. We have corrected this issue. |  |
| Avoid using p-value when the finding is not significant.  p 17 lines 362-363 a reference seems missing while reference 26 is unnecessary | Sorry for typing error. We have corrected this issue. |  |
| p 17 lined 369-371: this phrase is in contrast with the results of the study and not clear | This part was deleted and the discussion was rewritten. | Discussion page 16 |
