# Supplementary material for: Comparison of whole-body muscle imaging findings between GNE myopathy and other young adult-onset hereditary myopathies
Source: PLoS One. 2026 Jan 23;21(1):e0341031. doi: 10.1371/journal.pone.0341031 (PMC12829776; doi:10.1371/journal.pone.0341031)
Supplement: Attachment — Submitted filename: Response_to_reviewer_auresp_2.docx [file pone.0341031.s005.docx]

Response to reviewer’s comments

Dear Editor,

We thank you and the reviewers for your thoughtful suggestions and insights. The manuscript has benefited from these insightful suggestions.

The manuscript has been rechecked and the necessary changes have been made in accordance with the reviewers’ suggestions. The responses to all comments have been prepared and given below. The responses are also indicated in the manuscript.

| Reviewer’s comments | Response | Change in the manuscript |
| --- | --- | --- |
| The authors made significant improvement to the manuscript that now sums up the main finding.  However, I suggest to double check the English translation in the tracked change text as some concepts are not clear. | | |
| pg.3 “ some patients, especially in case of longstanding inconclusive diagnosis, may present with late stage which all muscle were involved and the patient may not recognize the sequential symptoms accurately” | This part was edited as the following “Although GNE myopathy had a recognizable clinical course showing early foot drop followed by proximal arms and legs weakness, the diagnosis of the GNE myopathy patients who presented with late stage of the disease is challenging as all muscles are affected and the clinical features mimics other hereditary myopathies.” | Introduction |
| In the abstract, the conclusion are not supported by the results (anterior forearm, biceps femoris involvement is not enumerated in the results section of the abstract. Moreover, anterior leg involvement is not even a finding of this study as “The soleus and tibialis anterior were severely affected in the early stages” (pg 14 and pg 25 line 525) | The abstract was corrected as the following: “Conclusion: The latissimus dorsi sparing out of proportion to periscapular weakness would be a novel differentiative feature of GNE myopathy.” | Abstract |
| vastus intermedius instead of intermedialis (e.g. Fig 1) | This typo was corrected. |  |
| please replace decimal commas with point throughout the text (e.g. pg 15) | In terms of comparing the parameters, the commas were used in bracket showing the interquartile value [Q1, Q3]. For example, “In the shoulder regions, latissimus dorsi in the GNE myopathy group had the lower median [IQR] fatty tissue replacement grade in T1WI (1 [0,1] vs. 3 [1,3.4], p=0.04)”. I do apologize for lacking of the space between commas and the Q3 value which mislead as a decimal comma. All of these typos were corrected. | Result section  Subheading - Comparison of T1WI and mDIXON Quant imaging between GNE and young adult-onset hereditary myopathies group |
| pg 23 line 488 typo “several ltechnical imitations were considered”—> several technical limitations… | This typo was corrected. |  |
| pg 26 line 547 and pg 26 line 566 typo “LGDM” —> LGMD | This typo was corrected. |  |
| pg 26 line 551 typo “LGMDR36” —> LGMDR3-6 | This typo was corrected as “LGMDR3-6 (sarcoglycanopathy)”. |  |
